# Past, Present and Future of Epigenetics in Adrenocortical Carcinoma

**DOI:** 10.3390/cancers12051218

**Published:** 2020-05-13

**Authors:** Madeleine Ettaieb, Thomas Kerkhofs, Manon van Engeland, Harm Haak

**Affiliations:** 1 Department of Internal Medicine, Division of Endocrinology, Maxima Medical Center, 5631 Eindhoven/Veldhoven, The Netherlands; H.Haak@mmc.nl; 2 Department of Internal Medicine, Division of Medical Oncology, Maastricht University Medical Center, 6229 Maastricht, The Netherlands; thomas.kerkhofs@mumc.nl; 3 Department of Pathology, GROW-School for Oncology and Developmental Biology, Maastricht University Medical Center, 6229 Maastricht, The Netherlands; manon.van.engeland@mumc.nl; 4 Department of Internal Medicine, Division of General Internal Medicine, Maastricht University Medical Center, 6229 Maastricht, The Netherlands; 5 Department of Health Services Research and CAPHRI School for Public Health and Primary Care, Maastricht University Medical Center, 6229 Maastricht, The Netherlands

**Keywords:** adrenocortical carcinoma, epigenetics, DNA methylation

## Abstract

DNA methylation profiling has been suggested a reliable technique to distinguish between benign and malignant adrenocortical tumors, a process which with current diagnostic methods remains challenging and lacks diagnostic accuracy of borderline tumors. Accurate distinction between benign and malignant adrenal tumors is of the essence, since ACC is a rare but aggressive endocrine disease with an annual incidence of about 2.0 cases per million people per year. The estimated five-year overall survival rate for ACC patients is <50%. However, available treatment regimens are limited, in which a radical surgical resection is the only curable option. Nevertheless, up to 85% of patients with radical resection show recurrence of the local disease often with concurrent metastases. Adrenolytic therapy with mitotane, administered alone or in combination with cytotoxic agents, is currently the primary (palliative) treatment for patients with advanced ACC and is increasingly used in adjuvant setting to prevent recurrence. Prognostic stratification is important in order to individualize adjuvant therapies. On April 1, 2020, there were 7404 publications on adrenocortical carcinoma (adrenocortical carcinoma) OR adrenocortical carcinoma [MeSH Terms]) OR adrenal cortex cancer[MeSH Terms]) OR adrenal cortical carcinoma [MeSH Terms]) OR adrenal cortex neoplasm [MeSH Terms]) OR adrenocortical cancer [MeSH Terms]), yet the underlying pathophysiology and characteristics of ACC is not fully understood. Knowledge on epigenetic alterations in the process of adrenal tumorigenesis is rapidly increasing and will add to a better understanding of the pathogenesis of ACC. DNA methylation profiling has been heralded as a promising method in the prognostication of ACC. This review summarizes recent findings on epigenetics of ACC and its role in diagnosis, prognosis and therapeutic strategies.

## 1. Introduction

Adrenocortical tumors (ACTs) are frequently discovered as incidentaloma due to increased use of imaging in a variety of medical settings. The first computed tomography (CT) series published in the early 1980s showed a prevalence of adrenal incidentaloma between 0.7–1.3% [1,2]. In a series published between 1982 to 1994 the mean prevalence of adrenal incidentaloma was 0.64% (ranging from 0.35–1.9%) [3], whereas, in 1991 Herrera et al. showed that only 0.4% of all the CT scans showed serendipitously discovered adrenal masses [4]). Bovio et al. showed a prevalence of 4.4% in their series of high resolution CT scans [5]. 

Most of these incidentalomas are benign adrenocortical adenomas (ACA), non-functional and clinically irrelevant. Their malignant counterpart, adrenocortical carcinoma (ACC), is a rare and aggressive type of cancer. Although estimates varied widely, the frequency of primary adrenal carcinoma in patients with adrenal incidentaloma ranges from 1.2–11% [6]. It should be kept in mind that due to the nature of these studies, selection bias is very probable (the populations studied not reflecting a random sample of all patients with an adrenal incidentaloma) and most likely leads to an overestimation of the frequency of ACC. Differentiating between these two types of tumors can be challenging, considering that clinical, laboratory, radiological and even histological features may overlap. Although ACC occurs in children (only 0.2% of pediatric cancers [7], annual incidence of 0.2 to 0.3 cases per 1 million individuals [8]), most cases appear between ages 30 and 50 (0.02% to 0.2% of adult cancers [9], annual incidence of 0.5–2.0 patient per million people per year [10]. An exception to these epidemiologic data is described in southern Brazil, where the annual incidence of adrenal cancer in children is unusually high, ranging from 3.4–4.2 per million children [11]. The distribution of tumors seems to follow a regional rather than familial pattern, therefore environmental factors have been considered, but so far none have been identified.

### 1.1. Molecular Alterations

**Figure 1:**
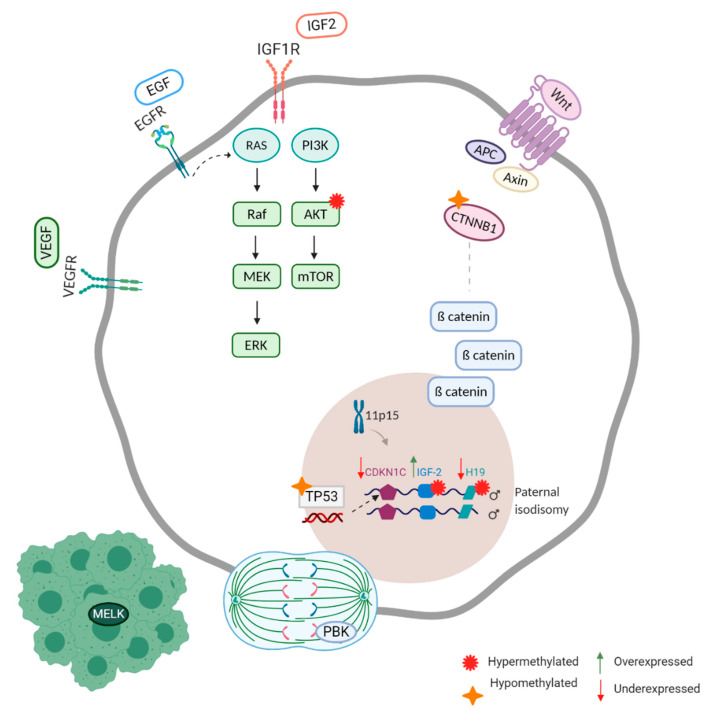
Major dysfunctional molecular pathways in adrenocortical carcinoma, resulting in abnormal survival, proliferation, apoptosis resistance, metastasis and angiogenesis [Created with BioRender.com].

The understanding of adrenocortical tumorigenesis has been challenging since molecular studies on adrenocortical carcinoma have been based on a small number of samples. Until recently they have been directed mainly to candidate genes. It was with this approach that the first genetic studies on ACC started with the elucidation of rare genetic syndromes (e.g. Li Fraumeni syndrome, Beckwith Wiedemann syndrome) in which ACTs are a manifestation [12]. They have led to the discovery of major dysfunctional molecular pathways in adrenocortical tumors, such as the IGF pathway, the Wnt pathway and TP53 (Figure 1) [12]. TP53 germline mutations have been described with a mutation prevalence of 3.9% [13].

Gene expression analysis showed that *IGF2*, a fetal growth factor imprinted at chromosome 11p15 locus, is upregulated and overexpressed in ACC. Another gene at the 11p15 locus, *H19* (a non-protein coding RNA) associated with the inhibition of *IGF2* expression, is under expressed in ACC [14]. 

In ACC, the Wnt/β-catenin (*CTNNB1*) is frequently activated trough *CTNNB1* mutations and even associated with a poor outcome [15]. In approximately 25% of both benign and malignant sporadic adrenocortical neoplasms β-catenin gain-of-function mutations are evident [16]. Zheng et al (2016) found 41% of ACC cases to have alterations of ZNRF3, CTNNB1, APC and MEN1 resulting in modification of the Wnt/β-catenin pathway [17].

Tyrosine-kinase coupled receptors have been confirmed to be abnormally active in the IGF pathway, the epidermal growth factor (EGF), fibroblast growth factor (FGF) and vascular endothelial growth factor (VEGF) pathway. These pathways are associated with cell survival, proliferation, angiogenesis, apoptosis resistance and metastasis [12]. 

Recently, efforts have been made to overcome the problem of small number of ACC samples by developing new preclinical models (CU-ACC1 and CU-ACC2) to advance ACC research [18]. For a long time H295R, initially established in 1980 from a 48-year-old female patient diagnosed with ACC, was the only cell line available for research [19]. With these new preclinical models Kiseljak-Vassiliades et al. have attempted to find new therapies by focusing on identifying the cell cycle kinases in ACC and pinpointing defects in the DNA damage response pathway. They analyzed publicly available expression data sets and observed that maternal embryonic leucine zipper kinase (MELK) was one of the most upregulated kinases in adrenal cancer compared to normal tissue [20].

This research group also observed the mitotic PDZ-binding kinase (PBK; also known as T-lymphokine-activated killer cell-originated protein kinase (TOPK)), a master mitotic kinase known for its role in mitotic division and regulation, to be highly overexpressed in ACC tissues compared to normal adrenal samples [21].

### 1.2. Prognostication

Clinical behavior among ACCs is heterogeneous and stage dependent. The extent of the disease at the time of diagnosis is best assessed by the European Network for the Study of Adrenal Tumor (ENSAT) staging score (Table 1) [22]. 

Prognosis in stage IV disease can vary from a few months to many years [23]. The immunohistochemical Ki67 index < 10% has been correlated with a relatively good prognosis where an index over 20% correlates with a more grim prognosis [24]. Despite the high inter- and intra-observer variability [25], Ki67 is currently the most accepted and used prognostic parameter [26] as confirmed by five studies [27,28,29,30,31]. It is recommended in the guidelines to use the Ki67 immunohistochemistry for every resection specimen of an adrenocortical tumor, and therapeutic strategies are suggested based on low-risk (Ki67 ≤10%) or high-risk (Ki67 >10%) stratification [32]. However, the ACC guideline does not provide directions for a standardized pre-analytical process or scoring approach for Ki67. There is a need for new reliable and reproductive diagnostic tests that can add to current classification scores or even outperform them and correlate better with biological behavior. It is currently not possible to predict whether a patient is cured after complete surgery commonly resulting in the prescription of adjuvant mitotane although some patients may not benefit from it. Over 20% of patients with tumor stages I to III die within the first 3 years, opting for a more aggressive post-operative systemic treatment. Furthermore, long-term survival of patients with metastatic disease at diagnosis has been described [23]. It is relevant to understand which patient with stage IV disease at primary diagnosis should be offered a treatment strategy facilitating such a long-term survival, or for which patients therapy should be aimed at quality of life and comfort [22]. 

Genomic profiling approaches have proven to be able to differentiate between adenomas and carcinomas and also to differentiate between carcinomas with different clinical outcome [33,34]. De Reynies et al. specified a malignant molecular cluster C1A and C1B versus a benign molecular cluster C2. Tumors of the C1A group had a very poor outcome and were enriched in transcription and mitotic cell cycle genes, whereas the good prognosis malignant C1B group was enriched in cell metabolism, intracellular transport, apoptosis and cell differentiation genes [34]. Giordano et al. showed a cluster analysis of the ACCs which revealed two subtypes that reflected tumor proliferation, as measured by mitotic counts and cell cycle genes [33]. Such accurate distinction is essential since treatment is radically different between adenoma and carcinomas. We were unable to find a study that compared a genomic profile versus regular immunohistochemical classification to differentiate between adrenal adenoma and carcinoma, therefore it is not clear whether genomics is superior to pathology. 

Giordano showed that pathologists accurately classify the large majority of ACTs using traditional clinicopathologic techniques, yet concluded that occasionally ACTs pose diagnostic challenges and would benefit from additional approaches and tools [35]. Lippert et al. found an improved prognostic stratification when implementing a modified Grading (G; ENSAT and Ki67), resection status (R), Age (A) and as tumor- or hormone-related symptoms (S) (mGRAS) system, by recognizing four ACC subgroups with a different clinical outcome by merging mGRAS and a molecular score (number of somatic mutations, alterations in the Wnt/b-catenin and p53/Rb pathways and promoter region methylation pattern) into a combined (COMBI) score. This molecular profile further improves the progression risk stratification identifying a group of patients with a favorable prognosis [36]. When superiority of COMBI with respect to mGRAS score was tested by discriminating patients with the best clinical outcome (at least 24 months free of disease progression).

COMBI score showed a better prognostic performance, proven by superior specificity (58.6% vs. 31.0%) and accuracy (83.3% vs. 74.5%). Also, when evaluating the disease-free survival (DFS) in a selected group of patients with ACC who were successfully operated (R0). In this subgroup, only COMBI score was able to identify a category of patients with an extremely longer DFS [36]. However, molecular profiling is not part of the European Guideline on ACC [32]. In addition, genomic studies lead to better understanding of tumor biology and hopefully could yield new insights to develop new therapies where current therapeutic options are limited, and available (chemo) therapies of limited effectiveness [37]. 

Over the last decade the understanding of adrenocortical tumorigenesis has improved and recently studies started focusing on epigenetic changes associated with adrenocortical tumors. Global DNA hypomethylation is a thoroughly studied example of an epigenetic alteration which is a hallmark of both benign and malignant tumors with unique methylation patterns [38]. Epigenetic alterations occur frequently in cancer cells and have the ability to mimic the effects of the latter [14,39,40]. Such epigenetic alterations are becoming increasingly accessible to analyze for an individual patient, and would be an interesting layer of additional molecular information to existing clinicopathological methods. Additionally, Feinberg (2018) suggested that differences in tumor types are related to the tissue of origin and often to the spectrum of mutations associated with that organ, whereas properties of the tumor heterogeneity and therapeutic resistance are epigenetic and are shared among tumor types. Therefore, understanding epigenetic regulation in cancer in general could provide valuable information needed to improve therapeutic strategies [41]. 

## 2. Genome Wide DNA Methylation

Rechache et al. [42] were the first to report a genome-wide DNA methylation profiling study in adrenocortical tumors: 19 normal, 48 benign, eight primary malignant and 12 metastatic malignant ones. They found, using the Infinium HumanMethylation450 BeadChips (Illumina, San Diego, CA, USA), that methylation patterns were distinctly different between normal, benign, primary malignant and metastatic tissue samples. Differentially methylated sites were found in both coding and noncoding regions of DNA. 

Interestingly, analysis of methylation patterns of benign adrenocortical tumor samples by functional status (cortisol secreting, aldosterone secreting, and nonfunctioning) showed different methylation patterns. Aldosterone-secreting tumor samples compared with nonfunctioning samples showed mostly hypomethylated CpG sites (75,3). There were only a small amount of differentially hypermethylated CpG sites between cortisol-secreting tumor samples compared with aldosterone-secreting tumor samples. No significant differences in the methylation pattern between cortisol secreting tumors and nonfunctioning tumor samples were found [42].This raises the question whether it is the methylation analysis that is unable to show difference between cortisol secreting tumors and nonfunctioning tumor samples or it is the clinical definition of ‘cortisol secreting’ and ‘nonfunctioning’. 

In cancer, hypomethylation usually occurs at repeated DNA sequences whereas hypermethylation predominantly involves CpG Islands [43]. This was also observed in ACC. Primary and metastatic ACC samples were globally hypomethylated compared to normal and benign samples. Hypermethylation in primary and metastatic ACC samples was predominantly seen in islands [42,44]. DNA methylation of the *H19* promoter has been shown to be involved in the abnormal expression of both *H19* and *IGF2* genes in the single gene study by Gao et al. (2002) [45]. Rechache et al. (2012) found 52 genes to be hypermethylated and downregulated in ACC (Table 2). Furthermore, of the differentially methylated genes in primary ACC, compared with benign tissue samples, several CpG sites were differentially methylated including those associated with *KCTD12*, *KRREL*, *SYNGR1*, and *NTNG2* and those in chromosome 11p15 imprinted region including *IGF2* and *H19*. Other sites were also in the *IGF2* pathway, including *IGF1R* that *IGF2* binds to and *AKT1*, a downstream signaling molecule in the cell survival pathway of *IGF1R*. *TP53* and *CTNBB1* both had hypomethylated sites, *RARRES2* and *SC16A9* had several hypermethylated sites in ACC tissue samples. 

Genetic studies have found IGF2 overexpression and *CDKN1C* and *H19* downregulation in 90% of ACC cases [33,50]. In pediatric ACC, *IGF1R* overexpression is associated with a worse prognosis. Genetic mutations of the *β-catenin* gene are common in preferentially non-functioning adenomas and in ACC [15,51]. 

Fonseca et al. [46] also analyzed the genome-wide methylation pattern of normal, benign and malignant adrenocortical tumors (Table 2). When comparing benign versus malignant ACT’s, they found that CpG islands were identified as significantly hypermethylated in ACC.

Primarily, genes involved in regulation of apoptosis, transcriptional, and cell cycle control showed significant and frequent hypermethylation. Only six genes known to be involved in the pathogenesis of other malignancies were further analyzed at mRNA level.

Expression of the significantly hypermethylated genes *CDKN2A*, *GATA4*, *DLEC1*, *HDAC10*, *PYCARD*, and *SCB3A1/HIN1* was reduced in both ACAs and ACCs compared to normal adrenal tissue. When treating the H295R ACC cell line in vitro with a demethylating agent (5-aza-2′-deoxycytidine), expression of all hypermethylated genes increased.

This was observed earlier in the study by Gao et al. in which *H19*, hypermethylated expression increased after treating the H295R ACC cell line with a demethylating agent [45]. These results suggest that epigenetic alterations could be reversible and a potential therapeutic target [49].

## 3. Candidate Gene Approach

Since *IGF2* is the most frequently overexpressed gene in ACC it is not surprising that single gene studies have focused on *IGF2*. Both Nielsen and Creemers studied differentially methylated regions patterns of *IGF2*. Three differentially methylated regions (DMRs) are involved in the regulation of *IGF2* expression. DMR0, DMR2 and the imprinting control region (ICR). *IGF2* DMR0 and DMR2 are located between exons 2 and 3 and exons 8 and 9 respectively (Table 3).

The *H19* and *IGF2* genes are separated from each other by the ICR. Nielsen found that 85% of the ACCs showed *IGF2* overexpression and *H19* down regulation, but did not found a correlation with clinical parameters such as the presence of metastases or TNM stage. The *H19* DMR is located upstream of the *H19* transcription start site. It harbors seven binding sites for the methylation-sensitive insulator CCCTC-binding factor (CTCF). 

Methylation status of the ICR is of direct influence on CTCF binding. CTCF only binds unmethylated ICR on the maternal chromosome. Upon binding, CTCF prevents communication between the proximal *H19* enhancer and the *IGF2* promoter, therefore keeping *IGF2* inactivated. CTCF cannot bind the paternal chromosome because the ICR is methylated. The enhancer is able to active *IGF2* transcription from the paternal chromosome. CTCF serves as a position-dependent insulator element to block inappropriate enhancer signals and protect against forged gene activation [58]. Creemers et al. (2016) found a significant change in methylation in CTCF3 and CTCF6 between ACC and ACA, where methylation in ACCs was higher [57]. Also, *H19* and *IGF2* showed significant hypermethylation in ACC [57]. 

Recently an increased interest has been raised for the relation between vitamin D and the adrenal gland [59]. Pilon et al. (2015) found methylation of the promoter of the vitamin D receptor and a reduced expression of the vitamin D receptor in ACC [56]. 

Earlier, inhibin alpha-subunit (INHA) was found to have a tumor suppressive role in adrenocortical tumorigenesis. In INHA knockout mice, 99% developed steroid-secreting ACCs after gonadectomy [60,61]. Hofland et al. (2014) investigated the methylation and expression of Inhibin α-subunit (encoded by INHA) in adrenal tumors. They found a significant difference in methylation of the INHA promotor between normal adrenals and ACCs. However, the promoter methylation in the ACC samples was not associated with tumor characteristics or ENSAT stage.

## 4. CIMP

It was in a study for colorectal cancer that the CpG island promoter methylation, or CIMP, was first discovered [62]. The term CIMP is controversial [63] since there is actually no universal standard or consensus with respect to defining CIMP. It is used to describe the increased prevalence of CpG island promoter methylation. So far this phenomenon has been described in multiple types of cancer: bladder, breast, endometrial, gastric, glioblastoma, hepatocellular, lung, ovarian, pancreatic, prostate, and renal cell cancers as well as in leukemia, melanoma and neuroblastoma. In 2012 Barreau et al. [47] analyzed CIMP in adrenocortical cancer. Tumor material of 135 patients with adrenal tumors was collected and clinical outcome was registered. ACCs were globally more hypermethylated than ACAs at the CpG islands in the promoter regions.

ACCs were clustered in two groups with different methylation levels. The first group carcinomas was slightly hypermethylated compared with adenomas, the second group was hypermethylated compared with both the adenomas and the carcinomas from the first group. The second group was again subdivided according to methylation level: a CIMP-high subgroup and a CIMP-low subgroup. The level of methylation was associated with survival and CIMP carcinomas show a worse prognosis compared to non-CIMP tumors.

Gene expression levels were increasingly down-regulated when comparing non-CIMP, CIMP-low and CIMP high carcinomas. These data suggest that differential methylation in the CpG promoter regions could be of clinical importance since they provide a classification based on methylation as a marker for prognosis in patients with adrenal tumors. 

Assié et al. (2014) [48] analyzed a cohort of 47 ACCs and an independent validation cohort of 77 ACCs, recruited from the European Network for the Study of Adrenal Tumors centers. Four DNA methylation-based tumor clusters were found. Two clusters corresponded to the CIMP-high and CIMP-low as described by Barreau et al. [47] associated with poor prognosis. Two other groups were categorized non-CIMP of which one showed widespread hypomethylation of CpG sites located outside CpG islands. 

miRNA expression was assayed and showed MIR483 to be overexpressed in ACC. MIR483 is located on the IGF2 locus, known to be involved in ACC. Based on miRNA expression levels Assié et al. (2014) identified three stable tumor clusters, Mi1-Mi3, with Mi1 having a significantly better overall survival rate then Mi2 and Mi3. Clusters were also established for mRNA expression in which two profiles were confirmed [34] to correlate strongly with survival, the aggressive C1A and indolent C1B. More importantly, a substantial overlap was found between the different omics of classifications. C1A (gene expression) with poor prognosis include almost all CIMP (DNA methylation) and Mi3 (miRNA expression) tumors. C1B tumors with a good prognosis were generally non-CIMP and belonged to the Mi1 or Mi2 mRNA clusters. 

The Cancer Genome Atlas (TCGA), an unique landmark cancer genomics program, began in 2006 and since then molecularly characterized over 20,000 primary cancer and matched normal samples spanning 33 cancer types (PubMed searched on 1 April 2020: 8422 citations of TCGA {search: "tcga"[Text Word]}. In 2016 Zheng et al. [17] analyzed 91 histologically confirmed adrenal tumors and matched it with tissue from a global cohort including 84 usual type, four oncoytic, two sarcomatoid, and one myxoid variant. Their pan-genomic approach yielded four mRNA-expression groups, six microRNA-expression groups, three copy-number groups, three protein-expression groups and three DNA-methylation groups. They integrated these ACC subsets through a cluster of clusters (CoC) analyses, resulting in three CoC subtypes, recognizing the fact that implementing four parallel profiling platforms poses a clinical challenge. The three methylation subtypes found (CIMP- low, intermediate and high) rendered discriminative representations of each CoC group, and classified the cohort into three ACC survival groups with 92.4% accuracy and were validated with an independent cohort.

Furthermore, it was shown that collectively the genes altered most frequently by somatic mutations, DNA copy-number alterations and epigenetic silencing were TP53 (21%), ZNRF3 (19%), CDKN2A (15%), CTNNB1 (16%), *TERT* (14%) and *PRKAR1A* (11%) [17].

## 5. Histone Modification

Histone modification is involved in the regulation of chromatin and gene expression. The best studied modifications are the acetylation and methylation of histones. Acetylation and deacetylation of histones is performed by histone modifying proteins such as histone acetyltransferases (HATs) and histone deacetylases (HDACs). At the transcriptional level, histone methylation is defined as the transfer of one, two, or three methyl groups from S-adenosyl-L-methionine to lysine or arginine residues of histone proteins by histone methyltransferases (HMTs). HMTs regulate DNA methylation through chromatin-dependent transcriptional repression or activation [64,65].

Drelon et al. performed a screen of histone methyltransferases, demethylases and associated factors in publicly available transcriptome data from ACC patients [66]. They observed the histone methyltransferase EZH2 to be overexpressed in ACC. High EZH2 expression levels, a result of deregulated P53/RB/E2f pathway, were associated increased cell proliferation/aggressive tumor behavior and poor prognosis in their study. 

Zheng et al. reported that 22% of their analyzed samples had dysregulated mRNA expression levels of histone modification genes *MLL*, *MLL2*, and *MLL4* and chromatin remodeling genes *ATRX* and *DAXX* [17]. Interestingly, seven percent of ACC cases have mutation in gene *MEN1*. *MEN1* encodes the tumour suppressor, menin, which has been reported to interact with HMTs *MLL*, *MLL2* [17,48].

## 6. Epigenetics and ACC Treatment

Genetic studies aimed at targeting biological pathways have not yet resulted in a significant breakthrough regarding therapeutic options. Inhibitors of both *IG2/IGF1R* and the mTOR pathways cause inhibition of cell proliferation of human ACC cell lines in vitro, and of growth of tumor xenografts in vivo [67,68]. A dual inhibitor of both *IGF-1R* and *IR*, linsitinib (OSI-906), was studied for the first time in humans in an open-label phase I study of 79 patients with advanced solid tumors, of which 15 patients had ACC. Although efficacy was not the primary end point of the study, two patients with ACC had partial responses [69]. Therefore, linsitinib versus placebo was studied in a double-blind placebo-controlled phase III trial. Unfortunately, linsitinib failed to show a difference in median overall survival (OS) or progression free survival (PFS) [70]. 

Also, everolimus, an mTOR inhibitor, showed no clinically meaningful response in patients with stage IV ACC [71]. Other attempts targeting VEGF and EGFR have also met with modest success [72,73]. Studies suggest that DNA methylation, in addition to genetic modifications causes altered patterns of gene expression resulting in tumorigenesis and harvest potential therapeutic markers [12,74,75].

A role for temozolomide (TMZ), a cytotoxic and antiproliferative agent, has been proposed in the treatment for ACC, which is thought to act primarily by alkylation of specific sites on especially the O^6^ position of guanine, which mispairs with thymine during the next DNA replication cycle [76]. The methyl group in O6-methylguanine can be removed by the O6-methylguanine-DNA methyltransferase (MGMT) gene, which leads to and impaired efficacy of TMZ. Epigenetic marks regulating MGMT expression are used as a predictive marker for response to TMZ in glioblastoma patients, since epigenetic silencing of MGMT sensitizes glioblastoma cells to TMZ [77]. Creemers et al. showed that ACC cell lines appear to have a low MGMT promoter methylation and observed a trend toward a slightly higher MGMT methylation in the responsive primary ACC cultures [76]. Curiously, overexpression of PBK/TOPK promotes the chemotherapeutic resistance to TMZ in glioma [78]. Both low MGMT promoter methylations as well as PBK overexpression could contribute to the observed short-lived control rate and poor prognosis in a clinical study with TMZ in 28 ACC patients [79].

Epigenetic targeted drug reports are still limited to in vitro ACC cell line studies. The study by Gao et al. was mentioned earlier: they treated H295R with Azad (also known as 5-aza/decitabine), a demethylating agent (5-aza-2′-deoxycytidine), which led to a significant increase in the H19 RNA content [45]. Decitabine is a drug which is currently approved by the Food and Drug Administration (FDA) for the treatment of myelodysplastic syndromes. It reverses the DNA promoter methylation.

Suh et al. also tested decitabine NCI-H295R cells. They observed a significant decrease in ACC cell proliferation by 39% to 47% at 5 days after treatment compared with control specimens (*p* < 0.001) [74]. Interestingly, decitabine has been shown to potentiate the cytotoxic effects of current chemotherapies, such as doxorubicin, cisplatin and etoposide, in neuroblastoma, suggesting that a combination of 5-aza with standard therapies could lead to more effective treatment [80].

Vorinostat was one of the first drugs to be approved that influence post-translational modification of histone proteins. Demeure et al. tested vorinostat in an ACC cell line which resulted growth inhibition. No studies on HMT inhibition were found. EHZ2 being overexpressed in ACC, HMT inhibition could be a potential treatment strategy in ACC.

Further research is required to determine the role of epigenetic targeted drugs in the treatment of ACC and overcoming drug resistance, where in other types of cancer epigenetic therapies are an emerging option for overcoming drug resistance [81]. 

Pan-genomic studies will initially contribute to the process of matching clinical/molecular profiles of patients with ACC with specific therapeutic programs and the understanding of therapeutic failures in the past. As Grisanti et al. noted, the COC1 cluster identified by Zheng et al. is characterized by a low grade of aneuploidy, better survival outcome, and high expression of the IGF2 pathway [17,82]. It could be considered that patients falling in this category would likely be more responsive to antieIGF2/IGF-1R compounds. Whereas, patients in the COC3 group characterized by mutations involving the cell cycle and DNA damage repair machinery would probably better respond to chemotherapy. Eventually the focus will shift to the understanding and identification of cancer dependencies based on functional genomic data and selection of priority drug candidates/drug repositioning in ACC.

Actually, Zheng et al. found 51 potentially actionable alterations in 22 ACCs, considering existing clinical trials and FDA-approved drugs for cancers [17]. Recently two excellent reviews have been published on this issue [75,83] which we encourage interested readers to consult.

## 7. Discussion

In cancer in general, there has been growing evidence to suggest that DNA methylation in addition to direct genetic modification causes altered gene expression resulting in tumorigenesis. Epigenetic analysis in adrenocortical tumors so far has been a significant addition to the understanding of molecular events involved in adrenocortical carcinogenesis. DNA methylation- based tumor clusters show overlap with other omics classifications. Clustering on epigenetic level allows differentiation between benign and malignant tumors and could be a significant addition to current histological parameters. Moreover, it might serve as an addition to current ENSAT staging in order to estimate prognosis and tumor aggressiveness. Currently no biomarker is included in the European Society of Endocrinology Clinical Practice Guidelines on ACC, but it is stated in the guideline that patients’ participation in registries and the collection of biological material as part of structured research programs aimed at defining biomarkers of diagnosis, prognosis and treatment response is encouraged [32].

**Figure 2:**
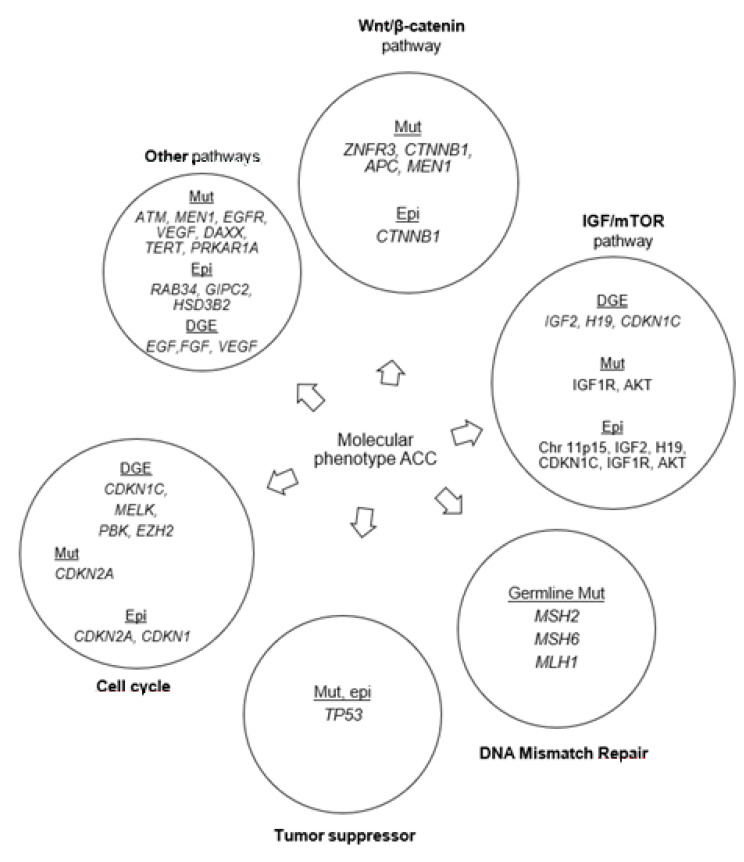
Frequently altered pathways in adrenocortical carcinoma as discussed in this review. Mut: mutations; DGE: differential gene expression; Epi: epigenetic modifications.

An interesting observation in the studies discussed above is the comparison between genetic and transcriptome-based studies (Figure 2). *IGF2* overexpression and structural abnormalities of 11p15 are present in up to 90% of cases of human ACC. *IGF2* expression is mediated by the insulin-like growth factor 1 receptor (IGF1R) which is also overexpressed in ACC. These genes have altered DNA methylation expression patterns in ACC. Zheng et al. found that 69% of tumours had at least one alteration of potential driver genes when combining somatic mutations, copy-number alterations, and epigenetic modification [17].

Further research is needed to understand the implications of epigenetic changes in adrenal tumorigenesis. Comprehensive data on well powered series are needed. For an orphan disease like ACC, united multinational consortia studies have the best chance of providing well powered data. 

Results of methylation associated gene expression levels in the articles discussed show heterogeneity (Table 2). For example, the 52 genes identified by Rechache et al. do not completely overlap with the top genes identified by Barreau et al. This discrepancy may be caused by methodological differences. Rechache et al. [42] used the 450 BeadChip, which provides a more comprehensive coverage of the genome with 17 times more CpG sites than the 27BeadChip that was used in the other studies. Nevertheless, when using Infinium 450k microarrays, data still is restricted to the particular genomic locations of the probes used in the array, which might not even necessarily capture the most relevant methylation sites. Koch et al. already advocated that a better understanding and more detailed analysis of the clinical relevance of the genomic location of DNA methylation is required to increase the number biomarkers that can be successfully implemented in patient care [84]. 

Also, it should be decided which platform of genomic analysis will be used in daily practice. Because, effectively, what is probably needed is a multi-omic molecular panel with the best selected biomarker predictors. 

DNA-methylation has proven to be replicable and able to provide accurate data on formalin-fixed or paraffin-embedded tumor samples [85,86] but no data has been published comparing the available genomic cluster entities in their ability to correctly diagnose adrenal malignancy and to predict recurrence, progression free survival and overall survival. Currently have been opted: C1A/C1B cluster [33,34,48] based on gene expressions, CIMP low/intermediate/high [17] cluster or CIMP low/high and non-CIMP [48] based on methylation profiles, CoC I-III as an integrated subset based on DNA copy number, DNA-methylation, mRNA-expression and miRNA-expression and Mi1-2 based on MiRNA expression. It is of importance to see how these clusters will perform when tested prospectively in a large cohort of adrenal tumors to validate these data and also establish the required cutoff values for the diagnosis of malignancy. Within these discriminating clusters, studies already are making an effort to identify markers representing these cluster sub-types. *G0S2* hypermethylation was shown to be a hallmark of the CIMP-high cluster [87]. When validated *G0S2* hypermethylation and the *BUB1B-PINK1* score could be potential markers on a molecular panel for ACC [34,87,88].

**Figure 3:**
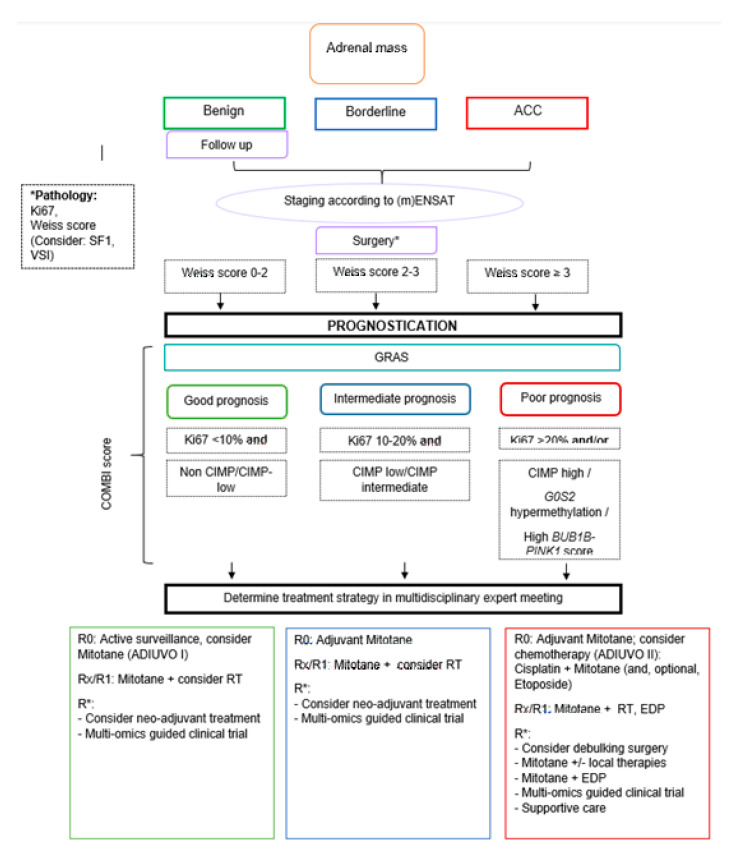
Flowchart on the potential management of the adrenal mass with the implementation of genomic analysis. Adrenocortical Carcinoma (ACC); Etoposide doxorubicin cisplatin (EDP); (modified) European Network for the Study of Adrenal Tumors ((m)ENSAT); Grade, Resection status, Age, Symptoms (GRAS); Complete resection (R0); Unknown radicality (Rx); Microscopically irradical (R1); ACC not amendable to radical resection (R*); Radiotherapy (RT); Steroidogenic factor-1 (SF1); Van Slooten Index (VSI).

Next steps will include the prospective comparison of the pathologic classification (Ki67 and Weiss score) of adrenal tumors versus a genomic assay versus the combination of both in the process of accurately diagnosing adrenal tumors. Evidence is needed that molecular data can improve the current diagnostic tools and that it does not matter whether it is genetic or epigenetic data. Finally, the bold step needs to be made to test the predictive value of these classifications in clinical practice by choosing a treatment regimen (Figure 3) based on the ACC prognostic cluster. Creemers et al. already showed that including *IGF2* methylation status to the pathology review could be supportive for the decision of adjuvant mitotane treatment [57]. Only after these steps, biomarkers could be officially implemented in the guidelines and acquire FDA approval.

Epigenetic changes may contribute to adrenocortical tumorigenesis by modulating size of the stem/progenitor population, altering phenotypic plasticity and enhancing sensitivity to subsequent mutations. ACC may develop in a multistep process. Therefore, it could be suggested that the level of DNA methylation is correlated with the risk of subsequent mutations, in which quantifying the influence of DNA methylation on gene expression remains difficult. Mutations in the *Wnt/β catenin* pathway have been shown to occur during progression [16,89]. Epigenetic and genetic mutations reflect alternate ways of inactivation during tumor progressions, i.e. a synergy between epigenetic and genetic alterations causing tumorigenesis, suggesting that combined inhibition of multiple affected pathways may hold the key to successful targeted therapy for ACC.

## 8. Conclusions

Research on adrenocortical tumors has been dominated by gene expression profiling and by analysis of genetic disorders associated with the predisposition of these tumors. With epigenetic studies, we are entering a new and complex phase in the understanding of ACC tumorigenesis. Analyzing the relationship between alterations in different layers of gene regulation could yield interesting insights. 

Finally, it will be challenging to not only use epigenetic analysis for diagnostic and prognostic purposes but also to keep investing in the development of new pharmacologic therapies and explore the potential of demethylating agents, because currently no significant therapeutic breakthrough is emerging. In the near future it will become interesting to see how the vast development of artificial intelligence, radiomics etc. will be of impact on diagnosis, prognosis and treatment of ACC.

## Figures and Tables

**Table 1:** ENSAT score.

Stage	TNM
I	T1,N0,M0 Tumor ≤5 cm
II	T2,N0,M0 >5 cm
III	T1–2,N1,M0 or T3–4,N0–1,M0
IV	T1–4,N0–1,M1

T, tumor. N, lymph node. M, metastasis. T1, tumor size ≤5 centimeter (cm); T2, tumor size >5 cm, T3, tumor infiltration in surrounding tissue; T4, tumor invasion in adjacent organs or venous tumor thrombus in vena cava or renal vein. N0, no positive lymph nodes; N1, positive lymph node(s). M0, no distant metastases; M1, presence of distant metastasis.

**Table 2:** Whole genome methylation studies on adrenocortical carcinoma.

**Study**	**Country**	**Year**	**N**	Population	19 NA; 47 Benign; 8 Primary malignant; 12 Metastatic malignant adrenals.
[42]	USA	2012	87	Method	Infinium HumanMethylation 450 BeadChips (Illumina, San Diego, CA, USA)
				Results	ACC show unique methylation patterns in which gene methylation status may be an important regulator of gene expression.
				Hypomethylated	TP53, β catenin (CTNNB1)
				Hypermethylated	↓ABCA1, CD55, CD74, COL4A3, GOS2, GATA6, **HSD3B2**, KCNQ1, MAP3K5, NCOA, RAPGEF4, RARRES2, S100A6, SPTBN1, TNFSF13, TNS1, ADCK3, ALDH3B1, CSDC2, CYP7B1, **GIPC2**, HOOK1, MEIS1, MLH3, MRPL33, NME5, RGNEF, TCIRG1, AMPD3, B4GALT6, CAB39L, GYPC, NDRG4, **RAB34**, RBPMS, SEMA6A, TNFS1F2-TNFSF13, SLC16A9, PHF11
				Diagnostic	‘Determination of the methylation difference in certain probe sites in ACT may be a useful diagnostic adjunct to histopathology for localized primary ACC.’
				Prognostic	-
				Therapeutic	**-**
[46]	USA	2012	48	Population	6 NA; 27 ACA (9 Nonfunctional,9 Cortisol producing, 9 Aldosterone producing); 15 ACC (9 Nonfunctional,6 Cortisol producing)
				Method	Infinium HumanMethylation27 Beadchip (Illumina, San Diego, CA)
				Results	CpG islands in the promoter regions are significantly hypermethylated in ACC.
				Hypomethylated	
				Hypermethylated	ZNF154, ALX4, ↓CDKN2A, GATA4, SCGB3A1/HIN1, PYCARRD, HDAC10 and DLEC1
				Diagnostic	**-**
				Prognostic	**-**
				Therapeutic	Treatment of ACC cell line H295R with 5-aza-2’-deoxytide showed significant restoration of gene expression of CDKN2A, GATA4, DLEC1, HDAC10, PYCARD and SCGB3A1/HIN1.
[47]	France	2013	135	Population	84 ACA; 51 ACC
				Method	Infinium HumanMethylation27 Beadchip (Illumina, San Diego, CA)MS-MLPA
				Results	ACC samples can be categorized according to CpG island methylator phenotype.
				Hypomethylated	
				Hypermethylated	↓H19, GSTM1, GSTP1, G0S2, GSTT1, **RAB34**, GYPC, **GIPC2**, PLAGL1, LY6D, PCOLCE, NDN, AMT, LGALS3BP, APOC1, TM7SF2, PPAPDC3, PTPN7, SCNN1A, **HSD3B2**, ACAA2, CTSZ, PYGM, KRT8, NDRG2
				Diagnostic	-
				Prognostic	The global level of methylation in CpG islands was associated with survival. CIMP carcinomas were associated with poorer prognosis.
				Therapeutic	-
[48]	France/	2014	81 **	Population	51 ACC; 30 ACA
	Europe			Method	Infinium HumanMethylation27 Beadchip (Illumina, San Diego, CA)
	(ENSAT)			Results	Confirmed CIMP in ACC. Tumor clusters based on different genomic approaches correlate.
				Hypomethylated	Nfs
				Hypermethylated	Nfs
				Diagnostic	-
				Prognostic	Transcriptome clusters were strongly correlated with DNA methylation clusters. The C1A subgroup with poor prognosis included almost all CIMP and Mi3 tumors. C1B tumors with good prognosis were generally non-CIMP and belonged to the Mi1 or Mi2 miRNA cluster.
				Therapeutic	-
[49]	USA	2015	116	Population	20 ACC; 75 Benign, 21NA
				Method	Infinium HumanMethylation 450 BeadChips (Illumina, San Diego, CA)
				Results	A cumulative comparison among gene methylation, copy number and miRNA profiling found that oncostatin M signaling, retinoic acid receptor activation (RXR) and PI3K/AKT and CDC42 signaling pathways were among the top pathways altered in ACC.
				Hypomethylated	
				Hypermethylated	TIPARP, RAPGEF4, RAB34, PPTC7, PDZRN3, OBSL1, NCEH1, MTMR6, METTL7A, LONRF2, LIMCH1, KLF9, KIAA1024, JAK1, ITGAV, ITGA2, **HSD3B2**, HLA-DPB1, DDC2, FOSL2, FGF12, FAMI198B, CYP1B1, CLU, CD59, CD55, C1QB, B4GALT6, IL13RA2, CDK1, ZMIZ1, TNS1, TBC1D4, SPTBN1, SLC16A9, SKAP2, SEMA6A, S100A6, RBPMS, RARRES2, RAB8B, PTPRG, PPP1R14A, NCOA7, MEIS1, MAP3K5, KCTD12, IL6ST, HTR2B, HOXA5, **GIPC2**, GATA6, G0S2, FSTL1, FMNL2, DDAH1, CD9, CD74, CD14, C3, C1RL, BNIP3L, AS3MT, APOC1, ABCA1, LPPR1, C9orf84
				Diagnostic	-
				Prognostic	-
				Therapeutic	Treatment of the ACC cell line, H295R, with decitabine (a global methylation inhibitor) increased the gene expression of CYP1B1 dramatically. It was found that oncostatin M inhibits ACC cell proliferation. Oncostatin M could inhibit ACC cell growth
[44]	USA	2016	24	Population	18 ACC (17 adrenal carcinomas, 1liver metastasis); 6 NA
				Method	Infinium HumanMethylation 450 BeadChips (Illumina, San Diego, CA)
				Results	It was demonstrated that ACC are globally hypomethylated compared to normal adrenal tissue. Hypomethylation was most frequent in ‘open seas’ and hypermethylation mostly in CpG islands. Epigenetic modulation of genes involved in TP53 stability and function, WNT signaling, and tumor suppressor genes were found.
				Hypomethylated	TMEM132D, ADCY2
				Hypermethylated	i.a. EPHX3, MEIS, CCDC8, TBX3, PAX8, DUSP7, DYRK2, RBM5, SETD7, NDRG1, UBE2D1
				Diagnostic	-
				Prognostic	-
				Therapeutic	-
[17]	USA	2016	79 ***	Population	91 adrenal tumors: 84 usual type, 4 oncocytic, 2 sarcomatoid and 1 myxoid variant.
				Method	Infinium HumanMethylation 450 BeadChips (Illumina, San Diego, CA)
				Results	Identified three Coc subtypes
				Hypomethylated	Nfs
				Hypermethylated	CDKN2A; Nfs
				Diagnostic	A methylation signature consisting of 68 probes robustly classified their cohort into three ACC survival groups with 92,4% accuracy.
				Prognostic	Coc analysis showed that molecular data can determine outcome with high significance.
				Therapeutic	-

Underlined results show overlap between two studies/Genes in **bold** were found to be hypermethylated in multiple studies. ↓ Under expressed genes. Adrenocortical Adenoma (ACA); Adrenocortical Carcinoma (ACC); Adrenocortical Tumors (ACT); CpG island promoter methylation (CIMP); Clusters of Cluster (Coc); European Network for the study of Adrenal Tumors (ENSAT); Normal adrenal (NA); Not further specified (nfs) ** [48] studied a total of 130 ACCs: 53 ACCs in their discovery cohort and 77 ACCs in their validation cohort. Only 51 samples from the discovery cohort were analyzed for DNA methylation profiling. ***. [17] only analyzed 79 samples for DNA methylation profiling.

**Table 3:** Single gene methylation studies on adrenocortical carcinoma.

**Study**	**Country**	**Year**	**N**	Population	16 NA; 10 ACC (2 Virilizing, 2 Nonfunctional, 6 Cushing’s); 16 ACA (2 Virilizing, 5 Cushing’s, 5 Conn’s)
[45]	Finland	2002	46	Gene	H19
				Method	Bisulfite-PCR
				Results	CpG sites in the H19 promoter are hypermethylated in ACC. IGFII is over expressed (methylation of IGFII not analyzed)
				Hypermethylated	H19
				Diagnostic	-
				Prognostic	-
				Therapeutic	ACC cell line NCIH295R was treated with Azad, a demethylating agent. It induced an increase in the H19 RNA content.
				Population	7 ACC; 8 ACA; 6 NA
[52]	USA	2013	21	Gene	RASSF1
				Method	Epitect methyl II PCR
				Results	There is a potential oncosuppressor role for RASFF1 in adrenocortical carcinogenesis.
				Hypermethylated	RASSF1
				Diagnostic	-
				Prognostic	All ACC showed reduced expression of RASSf1A, irrespective of their clinical characteristics or malignant stages.
				Therapeutic	-
				Population	39 ACA (16 Nonfunctional, 16 Aldosterone producing, Cortisol producing); 3 ACC; 23 NA
[53]	Japan	2014	65	Gene	Wif-1
				Method	MSP, USP & Bisulfite-PCR
				Results	57,1% of the adrenal tumours were found to be positive for Wif-1 methylation. No sub analysis specific for ACC.
				Hypermethylated	Wif-1
				Diagnostic	-
				Prognostic	-
				Therapeutic	-
				Population	3 NA; 19 ACC
[54]	Netherlands	2014	22	Gene	INHA
				Method	Bisulfite-PCR
				Results	A subset of ACCs has an increased methylation ratio of several CpGs in the INHA promoter.
				Hypermethylated	INHA
				Diagnostic	-
				Prognostic	No association with van Slooten index or ENSAT stage.
				Therapeutic	-
				Population	12 Conn’s; 10 Pheochromocytoma; 20 ACA, 20 ACC
[55]	France	2015	62	Gene	IGF2
				Method	Pyro-sequencing Bisulfite-PCR
				Results	IGF2 overexpressed in 85% of ACCs and 100% of PCC. Significant decreased expression of H19 in ACCs. 15/19 ACCs had somatic copy number alterations at the IGF2/H19 locus, with 6/15 having an extra copy of the allele.
				Hypomethylated	IGF2-DMR2
				Hypermethylated	H19-ICR (CTCF2, CTCF3, CTCF6); 3 CPGs of DMR0
				Diagnostic	3 CPGs of DRM0 correlated positively with the Weiss score.
				Prognostic	Expression levels of IGF2 did not correlate with clinical parameters such as presence of metastases or TNM stage. The presence of more paternal alleles than maternal alleles was significantly associated with the presence of metastases.
				Therapeutic	-
[56]	Italy	2015	26	Population	3 NA; 15 ACA (3 Nonfunctional, 10 Aldosterone producing, 2 Cortisol producing); 8 ACC
				Gene	VDR
				Method	Bisulfite-PCR
				Results	Methylation in the VDR promoter was observed in 3/8 ACCs. Methylation sites were identical in all 3 ACCs. No VDR promoter methylation was found in the other 5 ACCs, 3 NAs and 15 ACAs.
				Hypomethylated	Nfs
				Hypermethylated	Nfs
				Diagnostic	-
				Prognostic	-
				Therapeutic	VDR promoter methylation is mentioned as potential drug target in ACC.
[57]	Netherlands	2016	49 + 22	Population	Cohort (*n* = 49): 24 ACC; 14 ACA; 11 NA Validation cohort (*n* = 22): 9 ACC; 13 ACA
				Gene	IGF2
				Method	Pyro-sequencing Bisulfite-PCR
				Results	DMR0, DMR2 no significant differences between ACC and ACA. CTCF3, CTCF6 and H19 hypermethylated.
				Hypermethylated	CTCF3, CTCF6, H19
				Diagnostic	IGF2 expression, DMR2, CTCF3 and H19 showed a significant predictive value for the diagnosis of ACC.
				Prognostic	-
				Therapeutic	Treatment of three human ACC cell lines (H295R, HAC15 and SW13) with the demethylating drug AZA significantly decreased IGF2 expression and increased H19 expression.

Adrenocortical carcinoma (ACC); Differentially methylated regions (DMR); Imprinting Control Region (ICR); Methylation-specific PCR (MSP); Normal adrenals (NA) Pheochromocytoma (PCC); Not further specified (Nfs); Unmethylation-specific PCR (USP).
